# Molecular evidence of *Monocercomonas* and *Acanthamoeba* in the feces of captive reptiles

**DOI:** 10.1007/s00436-022-07677-3

**Published:** 2022-10-03

**Authors:** Barbara Tuska-Szalay, Hannah Kelly, Nóra Takács, Jenő Kontschán, Jan Votýpka, Sándor Hornok

**Affiliations:** 1 Department of Parasitology and Zoology, University of Veterinary Medicine, Budapest, Hungary; 2 Present Address: New Blood-Sucking Parasites and Vector-Borne Pathogens Research Group, ELKH-ÁTE Climate Change, Budapest, Hungary; 3 Plant Protection Institute, Centre for Agricultural Research, ELKH, Budapest, Hungary; 4 Biology Centre, Institute of Parasitology, Czech Academy of Sciences, České Budějovice, Czech Republic; 5 Department of Parasitology, Faculty of Science, Charles University, Prague, Czech Republic

**Keywords:** Protozoa, Trichomonadea, *Acanthamoeba*, Squamata, SSU rRNA gene

## Abstract

Reptiles are frequently kept as pet animals. They are considered as important reservoirs of protozoa with veterinary-medical significance. At a reptile farm in Ireland, fecal samples were collected from 98 captive reptiles, representing 43 species of three orders (Squamata, Testudines, and Crocodylia). After DNA extraction, all samples were screened by conventional PCRs, targeting the ribosomal small subunit (SSU) RNA and alpha-tubulin genes of trichomonads and SSU RNA gene of *Acanthamoeba* spp. One leopard gecko (*Eublepharis macularius*) was positive for a not yet reported species/genotype of the genus *Monocercomonas*, different from *M. colubrorum*. Various *Acanthamoeba* genotypes were detected in six reptilian species, i.e., *Acanthamoeba* genotype T11 in *Eunectes notaeus* and *Heloderma suspectum/horridum*; genotype T4 in *Varanus exanthematicus*, *Chlamydosaurus kingii*, and *Macrochelys temminckii*; and the genotype T13 in *Iguana iguana*. Some of these amoeba species might have clinicopathological significance in both humans and animals. Our findings highlight the importance to monitor pathogenic protozoa in pet as well as wildlife reptiles, as a source of possible infection for animals and humans living nearby.

**Supplementary Information:**

The online version contains supplementary material available at 10.1007/s00436-022-07677-3.

## Introduction

Nowadays, reptiles are increasingly considered as pet animals. This alone makes it important to study microorganisms that infect these vertebrates. Furthermore, in captivity, some of the reptile-associated pathogens could have significant veterinary and medical relevance for other pet and livestock animals or even humans (Mendoza-Roldan et al. 2020).

Mucosoflagellates are protozoa known for their flagella and for their dwelling on the surface of the mucous membranes. This group includes the phylum Metamonada and the clade Parabasalia among which species of the latter have parabasal body that is connected to their flagellar apparatus (Cepicka et al. 2016). This phylum has more genera, e.g., *Hexamita*, *Giardia*, *Monocercomonas*, and *Trichomonas.* Two of the latter belong to the order Trichomonadida and might be found in reptiles (Vilela et al. 2003; Rataj et al. 2011). They infect mainly the urogenital and gastrointestinal tracts of their host and have hitherto been detected in numerous reptile species, mostly based on morphological observations (Vilela et al. 2003; Corriveau and Thompson 2013). Members of the family Trichomonadidae were found in both snakes and lizards, e.g., in *Bothrops jararaca*, *Eryx johnii*, *Phelsuma dubia*, and *Physignathus cocincinus* (Vilela et al. 2003; Rataj et al. 2011). In addition, *Monocercomonas* sp. as a member of Monocercomonadidae family was detected in snakes and lizards causing depression, weight loss, and diarrhea (Zwart et al. 1984).

Among species that belong to phylum Amoebozoa, the opportunistic *Acanthamoeba* species phylogenetically belong to Acanthamoebidae family. The host of these species might be the reptiles (Sesma and Ramos 1989; Corsaro 2020); in addition, some of them can be pathogenic to domestic animals and also to humans (Geisen et al. 2014), causing skin lesions, keratitis (*Acanthamoeba* keratitis (AK)), or encephalitis (granulomatous amoebic encephalitis (GAE)) (Marciano-Cabral and Cabral 2003; Siddiqui and Khan 2012). These ubiquitous protists have been detected in the gastrointestinal tract, feces, brain, and skin lesions of reptiles (Schuster and Visvesvara 2004).

In this study, we screened the feces of a broad range of captive reptiles for DNA of protozoan parasites with veterinary-medical significance from the clade Parabasalia.

## Materials and methods

Samples of 98 reptiles were collected at the National Reptile Zoo in Kilkenny City, Ireland, between March and July 2021. These captive animals represented 43 species and belonged to three orders (Squamata, Testudines, and Crocodylia) (Supplementary Table 1). None of them has been purchased recently, and they did not show any clinical symptoms. From all of them, fecal samples were obtained in a non-invasive way; i.e., their fresh feces were collected from their artificial enclosure, attempting to exclude soil contamination. Fecal samples were placed inside pre-labeled Sarstedt tubes which were then stored at -20 °C until sample processing.

DNA was extracted using the QIAamp® Fast DNA Stool Mini Kit (QIAGEN, Hilden, Germany) according to the manufacturer’s instructions with some modifications (i.e., prior to adding Buffer AL, the solution was incubated at 56 °C for 60 min, and then the Buffer AW1 was used twice during the washing procedure). DNA extracts were stored at -20 °C until molecular analyses by conventional PCRs. For each PCR method, 5 µl of extracted DNA was added to 20 µl of reaction mixture containing 1.0 U HotStar Taq Plus DNA polymerase (5 U/µl) (Qiagen, Hilden, Germany), 0.5 µl dNTP Mix (10 mM), 0.5 µl of each primer (50 µM), 2.5 µl of 10 × Coral Load PCR buffer (15 mM MgCl_2_ included), and 15.8 µl distilled water. Further details of primers and PCRs are summarized in Technical Appendix. In all PCRs, sequence-verified positive controls were included.

The purification and sequencing of the PCR products were done by Biomi Ltd. (Gödöllő, Hungary). Obtained sequences were checked using the BioEdit program and then compared to GenBank sequences using the BLASTn program (https://blast.ncbi.nlm.nih.gov). The sequences obtained were submitted to GenBank (*Monocercomonas*, OM455397; *Acanthamoeba* spp., OM455398-OM455403). All sequences retrieved from GenBank were trimmed to the same length prior to phylogenetic analysis. This dataset was resampled 1000 times to generate bootstrap values. Phylogenetic analysis was conducted with the maximum likelihood method using MEGA 7.0 (Kumar et al. 2016).

## Results and discussion

**Fig. 1:**
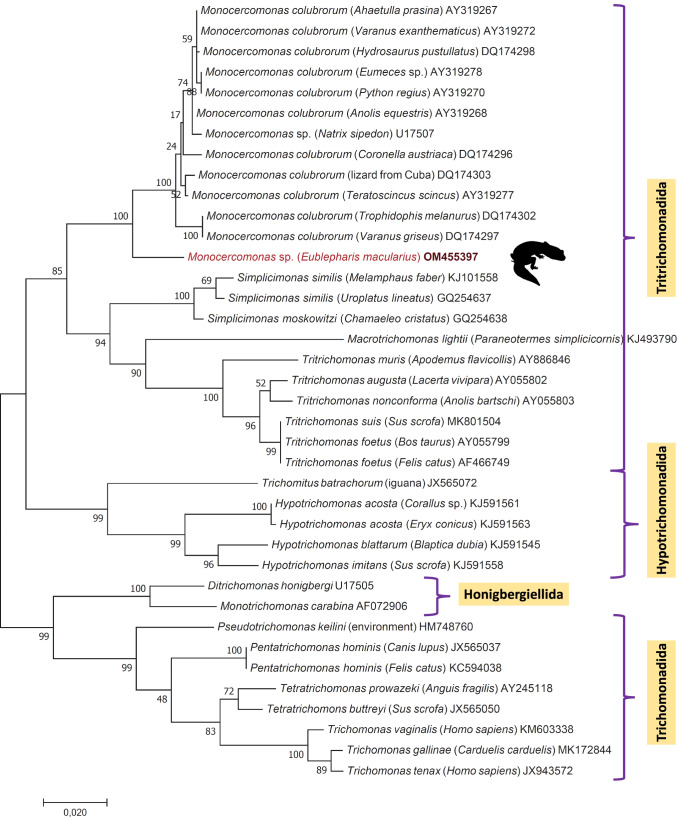
Phylogenetic tree of Trichomonadea. In each row, after the species or genus name, the isolation source and GenBank accession number are shown. Sequences obtained in this study are indicated by red fonts and bold accession numbers. The reptile species which had PCR-positive sample is shown with its silhouette. The evolutionary history was inferred by using the maximum likelihood method based on the general time reversible model [1]. The tree with the highest log likelihood (− 8331.32) is shown. The percentage of trees in which the associated taxa clustered together is shown next to the branches. Initial tree(s) for the heuristic search were obtained automatically by applying neighbor join and BioNJ algorithms to a matrix of pairwise distances estimated using the maximum composite likelihood (MCL) approach and then selecting the topology with superior log likelihood value. The tree is drawn to scale, with branch lengths measured in the number of substitutions per site. The analysis involved 38 nucleotide sequences. Codon positions included were 1st + 2nd + 3rd + noncoding. All positions containing gaps and missing data were eliminated. There were a total of 1402 positions in the final dataset. Evolutionary analyses were conducted in MEGA7.0

A single sample from a leopard gecko (*Eublepharis macularius*) was positive in the PCR targeting the ribosomal small subunit (SSU) RNA gene or 16S-like rRNA gene of trichomonads. Sequencing verified the presence of a not yet reported *Monocercomonas* genotype/species, with only up to 96.1% (1446/1505 bp) identity to the closest match available in GenBank (DQ174303) that represented *Monocercomonas colubrorum* (Hampl et al. 2007) and 94.9–95.9% (1432/1509 to 1443/1504 bp) identity to further sequences reported from this species and its genus. The phylogenetic separation of the new genotype from *Monocercomonas* sequences retrieved from GenBank, including those of *M. colubrorum*, was highly (100%) supported (Fig. 1). However, amplification of part of the alpha-tubulin gene was not successful from the *Monocercomonas*-positive sample.

Trichomonads and related mucosoflagellates are considered as nonpathogenic commensalists (Vilela et al. 2003), although in some cases they might cause loss of appetite, diarrhea, and weight loss of reptiles (Machin 2015). Occasionally, their establishment in the gallbladder results in cholangitis. The pathogenic role of some species from order Trichomonadida is supported by the fact that in a fecal sample of a viper (*Bothrops jararaca*) with diarrhea, high numbers of trichomonads were detected microscopically (Vilela et al. 2003). Furthermore, in a black throat monitor lizard (*Varanus albigularis ionidesi*), a coinfection with two protozoa, including *Trichomonas* and *Cryptosporidium* spp., caused diarrhea, salivation, vomiting, anorexia, and lethargy (Corriveau and Thompson 2013). *Trichomonas* species have been described as frequently found protozoa in reptiles of Ceylon (*Vipera russeli*, *Calotes versicolor*) (Kannangara 1970); however, with molecular investigations, it has not since been clarified which *Trichomonas* species are involved. Furthermore, *Monocercomonas* spp. are known to live in the large intestine and pass with the feces of squamate reptiles (Da Silva et al. 1998). They may cause moderate depression, loss of activity and weight (Zwart et al. 1984).

In the present study, a novel *Monocercomonas* genotype was found in a leopard gecko. *Monocercomonas colubrorum* is considered a common species in a wide range of Squamata (Moskowitz 1951; Hampl et al. 2007), but, to the best of our knowledge, no other species of this genus has hitherto been described from reptiles. Therefore, based on the sequence data obtained in this study it is possible, that our finding represents a new species or perhaps even a new genus. Considering phylogenetically it belonged to a sister clade of all other reported *Monocercomonas* sequences (Fig. 1).

**Fig. 2:**
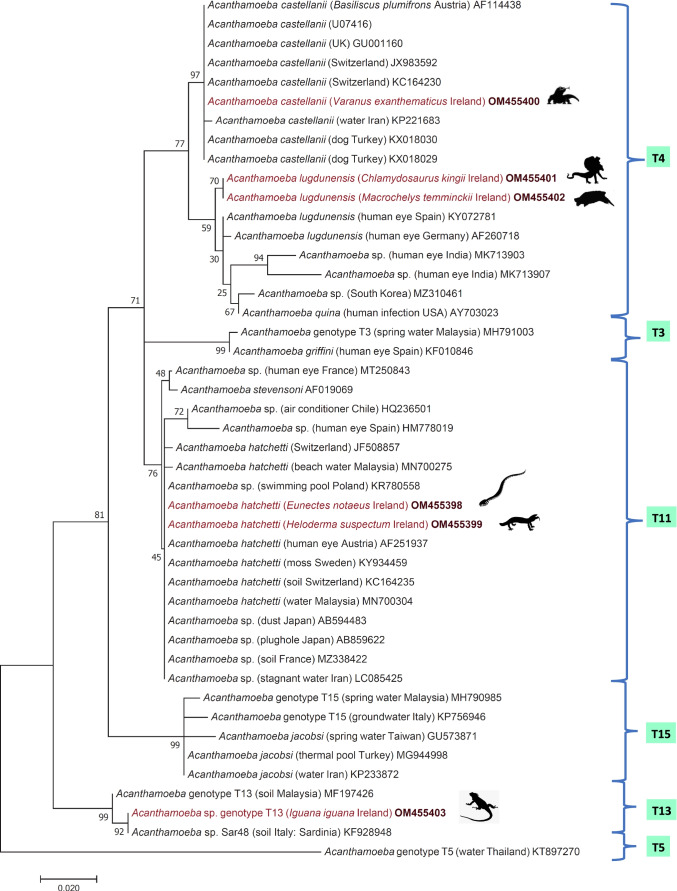
Phylogenetic tree of *Acanthamoeba* spp. In each row, after the species or genus name, the isolation source, the country of origin, and GenBank accession number are shown. Sequences obtained in this study are indicated by red fonts and bold accession numbers. Reptiles which had PCR-positive samples are shown with their silhouette. The evolutionary history was inferred by using the maximum likelihood method based on the Jukes–Cantor model. The tree with the highest log likelihood (− 8520.58) is shown. The percentage of trees in which the associated taxa clustered together is shown next to the branches. Initial tree(s) for the heuristic search were obtained automatically by applying neighbor join and BioNJ algorithms to a matrix of pairwise distances estimated using the maximum composite likelihood (MCL) approach and then selecting the topology with superior log likelihood value. The tree is drawn to scale, with branch lengths measured in the number of substitutions per site. The analysis involved 45 nucleotide sequences. Codon positions included were 1st + 2nd + 3rd + noncoding. All positions containing gaps and missing data were eliminated. There were a total of 404 positions in the final dataset. Evolutionary analyses were conducted in MEGA7.0

Fecal samples of six reptile species showed positivity in the PCR targeting SSU rDNA, which is specific for the genus *Acanthamoeba*, and all detected species/genotypes clustered with other *Acanthamoeba* sequences available in GenBank (Fig. 2). In two samples, one from a yellow anaconda (*Eunectes notaeus*) and the other from a Gila monster (*Heloderma suspectum*) or a beaded lizard (*H. horridum*) kept together, *Acanthamoeba hatchetti* was identified (OM455398 and OM455399, respectively), with 100% (408/408 bp) sequence identity to an isolate from compost in Switzerland (KC164235) (Conza et al. 2013). Based on the phylogenetic examination, they clustered with the strains of T11 genotype (Fig. 2). From a bosc monitor (*Varanus exanthematicus*), the amplified sequence (OM455400) had 100% (414/414 bp) identity to strains of *A. castellanii* (accession numbers KX018029, KX018030) detected in conjunctival swabs of dogs reported from Turkey (Karakuş et al. 2016). The sequence from a frilled dragon (*Chlamydosaurus kingii*, OM455401) and an alligator snapping turtle (*Macrochelys temminckii*, OM455402) showed 99.75% (406/407 bp) identity with *A. lugdunensis* (KY072781) from a human patient with keratitis in Spain (Martín-Pérez et al. 2017). Furthermore, the sample of a green iguana (*Iguana iguana*, OM455403) was 100% identical (404/404 bp) to *Acanthamoeba* T13 genotype (KF928948) from grassland soil, Italy (Geisen et al. 2014). In addition, phylogenetically, it grouped together with the strains of T13 genotype (Fig. 2).

Since reptiles are frequently kept as pet animals, the parasites they carry can be a source of infection for other animals or humans. Reptiles that are sold as pets might not born in captivity but brought from the wild, thereby increasing the chances of carrying different pathogens (Rataj et al. 2011). Opportunistic *Acanthamoeba* spp. are geographically widespread and locally live in diverse environmental substances, including soil, water reservoirs, and even hospitals (Geisen et al. 2014; Cooper et al. 2021). Although *Acanthamoeba* spp. play an important role in maintaining bacterial biomass (Geisen et al. 2014), they are of great public health significance because they can cause encephalitis (GAE), skin lesions in immunocompromised patients, and keratitis (AK) mainly in people wearing contact lenses (Marciano-Cabral and Cabral 2003; Siddiqui and Khan 2012). Furthermore, these species also have veterinary significance, as their presence is proven in animals, including dogs, cats, pigs, horses, rabbits, birds, amphibians, and reptiles (Siddiqui and Khan 2012). They may also have clinical significance in some animals, since conjunctival swabs of birds, dogs, and cats contained these protozoa (Cooper et al. 2021); however, in cats with keratitis, screening their corneal scrapings also proved to be effective (Ledbetter et al. 2021). Interestingly, in a study *Acanthamoeba* sp. from the eyes of cats showed 100% identity to *A. castellanii* genotype 4 from human eyes (Ithoi et al. 2013). In addition, *Acanthamoeba* sp. might also occur in the brain of animals, as it has been reported in a dog and in a rhesus macaque (Westmoreland et al. 2004; Dubey et al. 2005).

Although *Acanthamoeba* spp. are ubiquitous protozoa, considering our carefully performed non-invasive sampling, molecular evidence provided in this study attests the presence of four different genotypes of *Acanthamoeba* in the feces of reptiles. *Acanthamoeba hatchetti* is known to have clinicopathological significance in both humans and animals, e.g., it was detected in a horse with severe placentitis (Begg et al. 2014). In addition, *Acanthamoeba* genotype T4 species are also opportunistic pathogens both in humans and animals causing keratitis and/or encephalitis (Yu et al. 2004).

Beside the classification according to cyst size and shape (group I–III) (Marciano-Cabral and Cabral 2003), *Acanthamoeba* spp. based on the SSU rDNA can be divided into 23 genotypes (T1–T23) (Norouzi et al. 2021); however, most of the AK cases are caused by the T4 genotype (Jercic et al. 2019). In this study, an *Acanthamoeba* sp. clustering with several isolates reported as *A. castellanii* and two identical *A. lugdunensis* sequences belonged to the phylogenetic group of the T4 genotype that is frequently isolated from human clinical cases (Khan 2006) (Fig. 2). In previous reports, although *Acanthamoeba* species have been found in reptile feces (Frank and Bosch 1972) and in the gut contents of reptiles (Sesma and Ramos 1989), they have been molecularly detected only from a necrotic lesion of basilisk lizard (Walochnik et al. 1999). To our knowledge, this is the first time that *Acanthamoeba* spp. are reported by molecular methods in the feces of several species of captive reptiles. These results could be important for human health since reptiles are frequently kept as pet animals. Furthermore, the vector role of *Acanthamoeba* spp. is also notable, since these amphizoic free-living amoebae can harbor different pathogens (Siddiqui and Khan 2012).

In conclusion, molecular evidence is provided here for the presence of *Acanthamoeba* DNA in the feces of captive reptiles. Although fecal samples analyzed here were collected in artificial enclosures, it cannot be completely ruled out that our PCR exceptionally could have amplified contaminating or air-borne *Acanthamoeba*. Nevertheless, the above findings of opportunistic pathogens highlight the importance of monitoring protozoa and bacteria in the feces of pet reptiles as a source of infections for other animals and humans living nearby. Furthermore, these data could even have epidemiological relevance in natural ecosystems, e.g., when raw juice is made for human consumption from fruits that may have become contaminated with the feces of arboreal reptiles.

## Supplementary Information

Below is the link to the electronic supplementary material.

Supplementary file1 (DOCX 23 KB)

Supplementary file2 (PDF 63.3 KB)

## Data Availability

The sequences obtained in this study are deposited in GenBank (*Monocercomonas*, OM455397; *Acanthamoeba* spp., OM455398-OM455403). All other relevant data are included in the manuscript and the references or are available upon request from the corresponding author.
