# Supplementary material for: Molecular evidence of Monocercomonas and Acanthamoeba in the feces of captive reptiles
Source: Parasitol Res. 2022 Oct 3;121(12):3681–7. doi: 10.1007/s00436-022-07677-3 (PMC9653362; doi:10.1007/s00436-022-07677-3)
Supplement: Supplementary file 1 — Supplementary file1 (DOCX 23 KB) [file 436_2022_7677_MOESM1_ESM.docx]

Technical Appendix

Primers and details for conventional PCR methods used in this study.

| **Target taxon** | **Target gene** | **Primer name** | **Primer sequence (5'-3')** | **Amplicon length (bp)** | **Thermocycling profile** | **References** |
| --- | --- | --- | --- | --- | --- | --- |
| Trichomonadea | ssu rDNA | 16SL  16SR1 | TAC TTG GTT GAT CCT GCC  TCA CCT ACC GTT ACC TTG | 1550 | 95 °C for 5 min; 35× (95 °C for 40 s; 50 °C for 30 s; 72 °C for 1 min); 72 °C for 7 min | (Cepicka et al. 2005) |
| Trichomonadea | alpha-tubulin  (nested PCR) | AtubA (outer)  AtubB (outer)  AtubF1 (inner)  AtubR1 (inner) | RGT NGG NAA YGC NTG YTG GGA  CCA TNC CYT CNC CNA CRT ACC A  TAY TGY YWN GAR CAY GGN AT  ACR AAN GCN CGY TTN GMR WAC AT | 1200  1200 | 95 °C for 5 min; 35× (95 °C for 40 s; 55 °C for 30 s; 72 °C for 1 min); 72 °C for 7 min;  95 °C for 5 min; 35× (95 °C for 40 s; 45 °C for 30 s; 72 °C for 1 min); 72 °C for 7 min | (Edgcomb et al. 2001; Cepicka et al. 2006) |
| *Acanthamoeba* | ssu rDNA | JDP1  JDP2 | GGC CCA GAT CGT TTA CCG TGA A  TCT CAC AAG CTG CTA GGG GAG TCA | 480 | 95 °C for 5 min; 35× (95 °C for 35 s; 56 °C for 45 s; 72 °C for 1 min); 72 °C for 7 min | (Schroeder et al. 2001) |

**References**

Cepicka I, Hampl V, Kulda J, Flegr J (2006) New evolutionary lineages, unexpected diversity, and host specificity in the parabasalid genus Tetratrichomonas. Mol Phylogenet Evol 39:542–551. https://doi.org/10.1016/j.ympev.2006.01.005

Cepicka I, Kutisová K, Tachezy J, et al (2005) Cryptic species within the Tetratrichomonas gallinarum species complex revealed by molecular polymorphism. Vet Parasitol 128:11–21. https://doi.org/10.1016/j.vetpar.2004.11.003

Edgcomb VP, Roger AJ, Simpson AG, et al (2001) Evolutionary relationships among “jakobid” flagellates as indicated by alpha- and beta-tubulin phylogenies. Mol Biol Evol 18:514–522. https://doi.org/10.1093/oxfordjournals.molbev.a003830

Schroeder JM, Booton GC, Hay J, et al (2001) Use of subgenic 18S ribosomal DNA PCR and sequencing for genus and genotype identification of acanthamoebae from humans with keratitis and from sewage sludge. J Clin Microbiol 39:1903–1911. https://doi.org/10.1128/JCM.39.5.1903-1911.2001
