# Supplementary material for: Molecular evidence of Monocercomonas and Acanthamoeba in the feces of captive reptiles
Source: Parasitol Res. 2022 Oct 3;121(12):3681–7. doi: 10.1007/s00436-022-07677-3 (PMC9653362; doi:10.1007/s00436-022-07677-3)
Supplement: Supplementary file 2 — Supplementary file2 (PDF 63.3 KB) [file 436_2022_7677_MOESM2_ESM.pdf]

Supplementary Table 1. Taxonomical summary of reptile species sampled in this study.

order	suborder	(super)family	English name of species	Latin name of species	sample number
TESTUDINES	Cryptodira	Chelydridae	Alligator snapping turtle	*Macrochelys temminckii*	HK69
		Testudinidae	Leopard tortoise	*Stigmochelys pardalis*	HK2, HK10, HK17, HK53
			Red footed tortoise	*Chelonoidis carbonarius*	HK4, HK36, HK38, HK56, HK61, HK62, HK87
			Pancake tortoise	*Malacochersus tornieri*	HK7, HK96
			Yellow footed tortoise	*Chelonoidis denticulatus*	HK66, HK81
	Pleurodira	Chelidae	Roti Island snakeneck turtle	*Chelodina mccordi*	HK32
SQUAMATA	Geckota	Gekkonidae	Leopard gecko	*Eublepharis macularius*	HK33, HK34, HK37, HK98, HK99
			Crested gecko	*Correlophus ciliatus*	HK35, HK60, HK65, HK14
			Henkel's leaf-tailed gecko	*Uroplatus henkeli*	HK85
	Scincomorpha	(Scincoidea)	Blue tongued skink	*Tiliqua scincoides*	HK15, HK8
		Gerrhosauridae	Sudan plated lizard	*Gerrhosaurus major*	HK3, HK71, HK76
		Teiidae	Black and white tegu	*Salvator merianae*	HK40, HK49, HK88
	Anguimorpha	Varandiae	Bosc monitor	*Varanus exanthematicus*	HK24, HK45
			Rough-necked monitor	*Varanus rudicollis*	HK63
			Asian water monitor	*Varanus salvator*	HK11
		Anguidae	Sheltopusik	*Pseudopus apodus*	HK82
		Helodermatidae	Gila monster	*Heloderma suspectum**	HK23, HK75, HK97 (*housed together)
			Beaded lizard	*Heloderma horridum**	
	Iguania	Iguanidae	Rhinoceros iguana	*Cyclura cornuta*	HK13, HK16, HK48, HK70, HK79, HK89
			Fiji banded iguana	*Brachylophus bulabula*	HK19
			Spiny tailed iguana	*Ctenosaura pectinata*	HK22, HK77
			Green iguana	*Iguana iguana*	HK52, HK5, HK72, HK88, HK91
			Casquehead iguana	*Laemanctus serratus*	HK68
			Desert iguana	*Dipsosaurus dorsalis*	HK93
		Agamidae	Indian garden lizard	*Calotes versicolor*	HK39, HK55, HK27
			Bearded dragon	*Pogona vitticeps*	HK9
			Frilled dragon	*Chlamydosaurus kingii*	HK6, HK8, HK25, HK42
			Australian water dragon	*Intellagama lesueurii*	HK18, HK57
			Striped water dragon	*Tropicagama temporalis*	HK54, HK50, HK44, HK59
			Asian water dragon	*Physignathus cocincinus*	HK94
			Sulawesi sailfin lizard	*Hydrosaurus celebensis*	HK73, HK90
	Serpentes	Boidae	Boa constrictor	*Boa constrictor*	HK30, HK12, HK31, HK86
			Yellow anaconda	*Eunectes notaeus*	HK51
			Colombian rainbow boa	*Epicrates maurus*	HK92
		Pythonidae	Royal python	*Python regius*	HK21, HK41, HK64, HK74
			Burmese python	*Python bivittatus*	HK26, HK67, HK80
			Carpet python	*Morelia spilota*	HK28, HK43
			Spotted python	*Antaresia maculosa*	HK84
		Colubridae	Milk snake	*Lampropeltis triangulum*	HK1
			California kingsnake	*Lampropeltis californiae*	HK20, HK29, HK78
			Gopher snake	*Pituophis catenifer*	HK46, HK83
		Elapidae	Monocled cobra	*Naja kaouthia*	HK95
CROCODYLIA	Eusuchia	Alligatoridae	Chinese alligator	*Alligator sinensis*	HK58
